# A Transient π–π or Cation–π Interaction between Degron and Degrader Dual Residues: A Key Step for the Substrate Recognition and Discrimination in the Processive Degradation of SulA by ClpYQ (HslUV) Protease in *Escherichia coli*

**DOI:** 10.3390/ijms242417353

**Published:** 2023-12-11

**Authors:** Chu-Hsuan Lin, Chih-Hsuan Tsai, Chun-Chi Chou, Whei-Fen Wu

**Affiliations:** 1 Department of Agricultural Chemistry, College of Bio-Resource and Agriculture, National Taiwan University, Taipei 10617, Taiwan; 2 Department of Microbiology and Immunology, College of Medicine, National Cheng Kung University, Tainan 701401, Taiwan

**Keywords:** SulA, ClpYQ ATP-dependent protease, degron, degrader, π–π interaction, cation–π interaction

## Abstract

The *Escherichia coli* ATP-dependent ClpYQ protease constitutes ClpY ATPase/unfoldase and ClpQ peptidase. The Tyr^91st^ residue within the central pore-I site of ClpY-hexamer is important for unfolding and translocating substrates into the catalytic site of ClpQ. We have identified the degron site (GFIMRP^147th^) of SulA, a cell-division inhibitor recognized by ClpYQ and that the Phe^143rd^ residue in degron site is necessary for SulA native folded structure. However, the functional association of this degron site with the ClpYQ degrader is unknown. Here, we investigated the molecular insights into substrate recognition and discrimination by the ClpYQ protease. We found that the point mutants ClpY^Y91F^Q, ClpY^Y91H^Q, and ClpY^Y91W^Q, carrying a ring structure at the 91st residue of ClpY, efficiently degraded their natural substrates, evidenced by the suppressed bacterial methyl-methane-sulfonate (MMS) sensitivity, the reduced β-galactosidase activity of *cpsB::lacZ*, and the lowest amounts of MBP-SulA in both in vivo and in vitro degradation analyses. Alternatively, mimicking the wild-type SulA, SulA^F143H^, SulA^F143K^ and SulA^F143W^, harboring a ring structure or a cation side-group in 143^rd^ residue of SulA, were efficiently degraded by ClpYQ in the bacterial cells, also revealing shorter half-lives at 41 °C and higher binding affinities towards ClpY in pull-down assays. Finally, ClpY^Y91F^Q and ClpY^Y91H^Q, were capable of effectively degrading SulA^F143H^ and SulA^F143K^, highlighting a correspondingly functional interaction between the SulA 143rd and ClpY 91st residues. According to the interchangeable substituted amino acids, our results uniquely indicate that a transient π–π or cation−π interaction between the SulA 143rd and ClpY 91st residues could be aptly gripped between the degron site of substrates and the pore site of proteases (degraders) for substrate recognition and discrimination of the processive degradation.

## 1. Introduction

In *Escherichia coli*, there are five AAA^+^ (ATPases associated with a variety of cellular activities) proteases, namely Lon, FtsH, ClpAP, ClpXP, and ClpYQ (HslUV) [1,2]. Uniquely, the two-component ClpYQ protease, consisting of ClpQ similar to a β subunit in the 20S proteasome and ClpY with an extra intermediate domain, was first found in *E. coli* [3,4,5,6]. According to bioinformatic, phylogenetic, structural, and functional analyses, ClpYQ is found in almost all eubacteria [5,7,8]. ClpQ (19 kDa) peptidase and ClpY (49 kD) ATPase/unfoldase are encoded by *clpQ^+^Y^+^* heat shock operons [3,4,6,9]. Using biochemical size-exclusion-chromatography, electronic microscopy, and X-ray crystal structure analysis, Y^6^Q^6^Q^6^Y^6^ was shown to exhibit a dumbbell-shaped complex with two ClpY and two ClpQ hexameric rings [5,10,11,12]. In the presence of ATP, ClpY is capable of delivering substrates into the catalytic ClpQ core site for degradation [6,13]. Hence, ClpY is responsible for substrate recognition, unfolding, and translocation with ATP hydrolysis [6,14,15,16,17]. Molecular dynamic multi-scale modeling demonstrates that the ClpYQ complex has a narrow central pore, and through it, only a single stretched peptide can pass [18]. However, another modeling study suggested that four-helix bundle protein substrates can be transported into the ClpQ core site for degradation with the ancillary ClpY-I domain [19].

From crystal structure analyses, the ClpY monomer constitutes three domains [12,20]. The N–terminal domain (N–domain, 2–109 aa and 244–332 aa) has ATPase activity; the pore I, II sites, and the intermediate–domain (I–domain, 110–243 aa) organize substrate binding; and the C–terminal domain (C–domain, 333–433 aa) allows for ClpY self-oligomerization and the activation of ClpQ catalytic function [6,12,16,17,20,21]. Inversely, ClpQ, with the substrate bound at the first threonine (Thr-1) catalytic residue, can allosterically activate the ATPase activity of ClpY-hexamer and increase its binding affinity toward ClpY [22]. Moreover, there are twelve potential active sites in the ClpQ dodecamer. However, only a maximum of six active sites are used for substrate degradation [23] and ClpQ demonstrates optimal proteolytic activity at pH 9 [24]. The GYVG^93rd^ amino acid sequence, especially the Tyr^91st^ residue, of pore site I central to the ClpY hexamer(s) is necessary for the binding/unfolding/translocation of substrates [15,16]. Moreover, two loops (130~159 aa and 175~209 aa) in the ClpY-I domain are involved in substrate delivery, in which three loop 2 unblock at higher temperatures, opening the route to the pore site for substrate degradation [6,17,25]. So far, several proteins have been identified as the cellular substrates for ClpYQ protease, including SulA, RcsA, RpoH, TraJ, RNaseR, YbaB and LpxC [26,27,28,29,30,31,32,33,34].

Both Lon and ClpYQ are capable of degrading SulA [28,29,33,34]. SulA functions as a cell division inhibitor by interacting with and inhibiting the activity of FtsZ, a critical GTPase essential for septum formation [35,36]. The tertiary structure of SulA comprises four α helices and five β sheets [37,38]. FtsZ individually binds to each monomer of the SulA dimer in a crystal structure [37] and has been shown to protect SulA from degradation by ClpYQ protease [34,39]. A recently identified degron site of SulA, GFIMRP^147th^, near the C-terminal was shown to be necessary for the recognition and binding of ClpYQ protease in degradation [38]. However, as of now, there is no reported crystal structure for the ClpY(6x)-substrate complex, and the mechanism underlying the recognition and discrimination of the degron site in natural substrates by the degrader for degradation remains unknown. Specifically, the Phe^143rd^ residue within SulA degron site has been found to be vital for folding the structure of SulA [38]. Thus, we suspected that these two residues, SulA-Phe^143rd^ and ClpY-Tyr^91st^, may be both involved in substrate denaturation and degradation at the molecular level.

Notably, the π–π interaction, an important noncovalent interaction responsible for π–electron interaction patterns, was progressively identified in the biological sciences [40]. Furthermore, the cation–π interaction, which represents another biological noncovalent interaction functioning within a limited distance between cations and adjacent π systems, has also presented its significance in biology [41]. Recently renowned studies have demonstrated that the amino acids with aromatic, imidazole or cation side chains in proteins for π–π interactions and cation–π interactions between the residues have been indexed for protein stability [42], protein–protein interaction [43], protein-ligand interactions [44], phase separation [45], catalysis [46] and protein assembly [40]. However, such interactions have not been reported for synthetic or natural substrate degradation by proteases. Here, by using site-directed mutagenesis to engineer protein molecules in physiological and functional-degradation assays, we explicitly demonstrate that a π–π or an amenable cation–π transient interaction between SulA-Phe^143rd^ residue in the degron and ClpY-Tyr^91st^ residue in the pore site of the degrader occurs for the substrate recognition and discrimination, and is necessary for the ultimate degradation of this natural substrate by the ATP-dependent protease.

## 2. Results

### 2.1. The Intracellular Degradation of Chromosomal RcsA and SulA by ClpYQ, ClpY^Y91F^Q, ClpY^Y91W^Q and ClpY^Y91H^Q

To investigate the underlined molecular mechanisms between SulA-Phe^143rd^ and ClpY-Tyr^91st^ residues, we constructed several ClpY variants bearing single substitutions of Y91F, Y91H, Y91K, Y91R, Y91S, and Y91W on the basis of both pBAD24-*clpY^+^* and His_(6x)_-tagged pET21a-*clpY^+^* plasmids. We also constructed SulA variants with point mutations of F143A, F143D, F143H, F143K, F143N, F143P, F143R, F143S, F143W and F143Y on the basis of both pTH18kr-*mbp-sulA^+^* and pMal-c2X-*sulA^+^* plasmids. In addition, new *E. coli* mutants were constructed in series as described in the Supplementary Materials (Figure S1, Table 1 and Table S1), including CH21408 (*lon*, *sulA*), CH21409 [(*lon*, *sulA*, *ftsZ*(SfiB^*^)], CH21410 (*lon*, *clpQ*, *clpY*, *sulA*) and CH21411 [(*lon*, *clpQ*, *clpY*, *sulA*, *ftsZ*(SfiB^*^)] strains.

**Figure 1:**
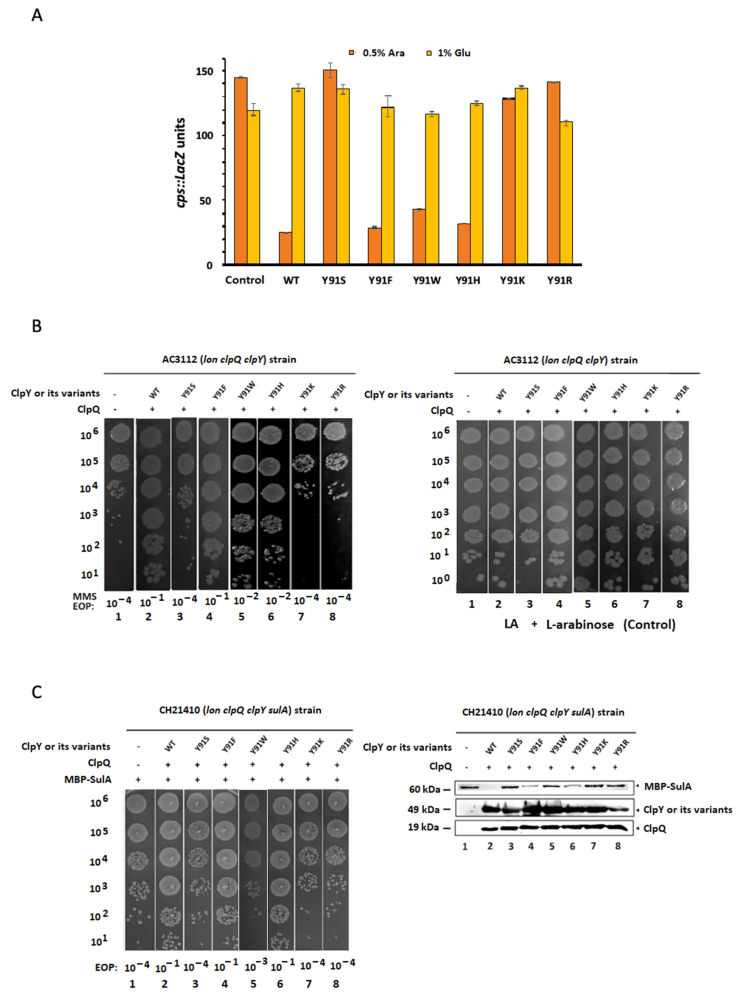
The β-galactosidase assays, methyl-methane-sulfonate (MMS) tests, the effects of MBP-SulA and the Western blot analyses. (**A**) AC3112 (*lon*, *clpQ*, *clpY*) bacterial cells carrying pBAD24-*clpY^+^* and its derivatives with pBAD33-*clpQ^+^* were grown in LB media, with appropriate antibiotics and 0.5% L-arabinose (induced condition) or 1% glucose (repressed condition). The bacterial cells, grown to OD_600_ = 0.5–0.9, were measured in series for β-galactosidase activity, to reflect *cpsB:lacZ* expression. (**B**) The log-phase bacterial cells grown in LB with 0.5% L-arabinose and appropriate antibiotics were diluted and spotted on LB-0.5% L-arabinose plates with 0.015% MMS (the left panel) or LB-0.5% L-arabinose plates without MMS (the right panel). The EOP value was determined through averaging the number of colonies counted on the growth media with an addition of 0.5% L-arabinose plus 0.015% MMS treatments, divided by the number of colonies counted on LB media plates containing 0.5% L-arabinose without MMS. (**C**) Left panel: CH21410 (*lon*, *clpQ*, *clpY*, *sulA*) bacterial cells carrying pBAD24-*clpY^+^* or its derivatives with pBAD33-*clpQ^+^* and pTH18kr-*mbp-sulA^+^* were grown in LB media, with 0.5% L-arabinose and appropriate antibiotics, to log phase at OD_600_ = 0.3–0.5 before adding IPTG (1 mM) for induction of MBP-SulA expression. After about 2 h of induction, half of the bacterial cells were diluted and spotted on LB plates with 0.5% L-arabinose. The EOP value was also determined via averaging the number of colonies counted on the growth media with an addition of IPTG, divided by the number of colonies counted on the growth media without IPTG. Bacterial cells carrying pBAD33, pBAD24 and pTh18kr-*mbp-sulA^+^* plasmids were used as negative controls. Right panel: Western blot analyses. The other half of bacterial cells after 2 h of induction were collected by means of centrifugation and re-suspended in 2X SDS-sample buffer. Next, in our Western blot analyses, equal amounts of the samples, normalized using recorded OD_600_ values, were loaded on 12.5% SDS-polyacrylamide gel for electrophoresis. Then, all the samples were transferred onto PVDF membranes (Thermo Scientific™, Waltham, MA, USA). Anti-MBP monoclonal antibody (mAb) was used for detection of the residual MBP-SulA proteins. ClpQ, ClpY and ClpY derivatives were detected using polyclonal ClpQ and ClpY anti-serum. Western blots were developed using an enhanced chemiluminescence (ECL) kit procured from Pierce.

Next, to explore the intracellular activities of wild-type ClpY and its derivative mutants in bacteria, an AC3112 strain (*lon*, *clpQ*, *clpY*) carrying pBAD33-*clpQ^+^* was used as the host, which was transformed individually with pBAD24-*clpY^+^* or its derivatives. An AC3112 strain carrying two empty vectors, pBAD24 and pBAD33, was used as a negative control. The resulting co-transformant carrying pBAD24-*clpQ^+^* and pBAD33-*clpY^+^* was then used as a positive control. The negative control AC3112 strain expressed higher β-galactosidase activity of *cpsB::lacZ* and was sensitive to methyl-methane-sulfonate (MMS) with a lower efficiency of plating (EOP) of 10^−4^, due to the reason that RcsA and induced-SulA were exceedingly stable in the absence of both Lon and ClpYQ proteases (Figure 1A,B). In contrast, similar to the wild-type ClpY, ClpY^Y91F^, ClpY^Y91W^ and ClpY^Y91H^ degraded RcsA and SulA proteins in the presence of ClpQ on L-arabinose media, as demonstrated by their host cells with lower β-galactosidase activity of *cpsB::lacZ* and a higher EOP (EOP ≧ 10^−2^) for the resistance to MMS (Figure 1A,B). However, the bacterial cells expressing ClpY^Y91S^Q, ClpY^Y91K^Q or ClpY^Y91R^Q did not degrade RcsA and SulA proteins due to their higher β-galactosidase activity and a lower EOP of 10^−4^ (Figure 1A,B). For additional confirmation of the aforementioned data, we transformed CH21410 (*lon*, *clpQ*, *clpY*, *sulA*) with pTH18kr-*mbp-sulA^+^* and pBAD33-*clpQ^+^*. The resultant bacterial cells were again transformed in series with pBAD24-*clpY^+^* and its derivatives. In Figure 1C, as our negative control, CH21410 bacterial cells carrying pBAD33, pBAD24 and pTH18kr-*mbp-sulA^+^* (a low copy plasmid) exhibited poor growth with an EOP of 10^−3^ after IPTG induction. However, we were able to rescue growth in CH21410 bacterial cells carrying pBAD33-*clpQ^+^*, pBAD24-*clpY^+^* and pTH18kr-*mbp-sulA^+^* after an IPTG induction for our auxiliary positive control. As shown in Figure 1C, in the presence of ClpQ, ClpY^Y91F^ and ClpY^Y91H^, like the wild-type ClpY, were also able to survive the intracellular lethality of MBP-SulA (EOP of 10^−1^). Interestingly, under similar conditions, bacterial cells carrying ClpY^Y91W^Q did not grow well (EOP of 10^−3^), and analogous results were found in bacterial cells carrying ClpY^Y91S^Q, ClpY^Y91K^Q or ClpY^Y91R^Q (EOP of 10^−4^). Coincidently, Western blot assays demonstrated that MBP-SulA was largely degraded by ClpYQ, ClpY^Y91F^Q and ClpY^Y91H^Q. However, an intermediary accumulation of MBP-SulA was detected in the bacteria with ClpY^Y91W^Q. In contrast, higher accumulation of MBP-SulA was significantly detected in the bacterial cells carrying either ClpY^Y91S^Q, ClpY^Y91K^Q or ClpY^Y91R^Q. These results indicate that ClpY mutants, with the aromatic residues, Phe (F) and Trp (W), as well as the imidazole ring residue, His (H), at the 91st position, retain their normal activity for natural substrate degradation in the presence of ClpQ.

### 2.2. The Distinct In Vitro MBP-SulA Degradation by ClpY, ClpY^Y91F^, ClpY^Y91H^ or ClpY^Y91W^ in the Presence of ClpQ and ATP

**Figure 2:**
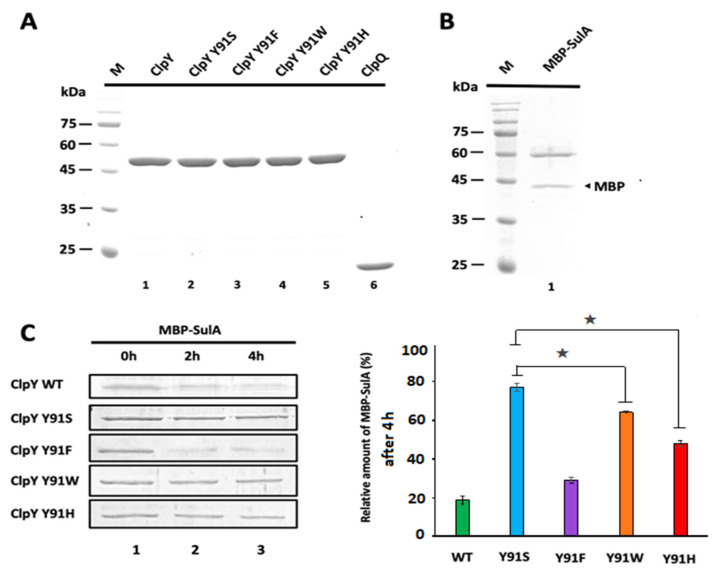
In vitro degradation of MBP-SulA by ClpYQ and its variant proteases. MBP-SulA, His (6x)-tagged ClpY, His (6x)-tagged ClpQ and their mutant variants were each expressed in *E. coli* BL21 (DE3) and purified. ClpQ, ClpY and its derivative proteins were each purified using His-tag affinity purification methods with cobalt chromatography, whereas MBP-SulA was purified using amylose resin (NEB), and the concentration of each protein was determined [16,17]. (**A**) ClpY (4 μM) protein, its variants (4 μM) and ClpQ (8 μM) separated by SDS-PAGE. M represents the protein marker. Lane 1: ClpY; Lane 2: ClpY^Y91S^; Lane 3: ClpY^Y91F^; Lane 4: ClpY^Y91W^; Lane 5: ClpY^Y91H^ and Lane 6: ClpQ. (**B**) M: the protein marker of SDS-PAGE. Lane 1 represents MBP-SulA (1 μM). (**C**) Degradation assays. Left panel: ClpY (4 μM) and its mutant proteins (4 μM) were each incubated with ClpQ (8 μM), MBP-SulA (1 μM) and ATP (5 mM) in the reaction buffer (50 mM HEPES buffer) at 41 °C and the samples were taken out at the time intervals of 0 h, 2 h and 4 h and subjected to SDS-PAGE. Right panel: The relative amounts of residual MBP-SulA, degraded within 4 h by ClpYQ or its derivatives, are plotted in the chart. Results were analyzed using Image J. The ★ asterisks indicate *p* < 0.05. Two-way Student’s *t*-test for single-pair comparison was used for statistical analysis.

We then conducted protein purification, as shown in Figure 2A,B. Purified ClpY protein and its derivative mutant proteins, including ClpY^Y91F^, ClpY^Y91S^, ClpY^Y91W^ and ClpY^Y91H^, were all tested for their ability to degrade the purified MBP-SulA in the presence of the pure ClpQ protein and ATP at 41 °C. Digestive mixtures were collected at 0 h, 2 h and 4 h and subjected to SDS-PAGE. Next, by using Image J analysis, relative levels of the remaining MBP-SulA in each sample were determined in the degradation assays. As noted in Figure 2C, within 4 h, ClpYQ and ClpY^Y91F^Q could both efficiently degrade MBP-SulA. In addition, ClpY^Y91H^Q exhibited moderate degradation activity. Meanwhile, ClpY^Y91W^Q revealed minor degradation activity, while ClpY^Y91S^Q retained the least degradation activity. These results again indicate that ClpY mutants, with a substituted ring amino-acid, Phe (F), His (H) or Trp (W), for the 91st residue, could still render the apparent degradation activity in the in vitro assays.

### 2.3. An Intracellular Degradation of MBP-SulA, SulA^F143H^, SulA^F143K^ and SulA^F143W^ by ClpYQ Protease

**Figure 3:**
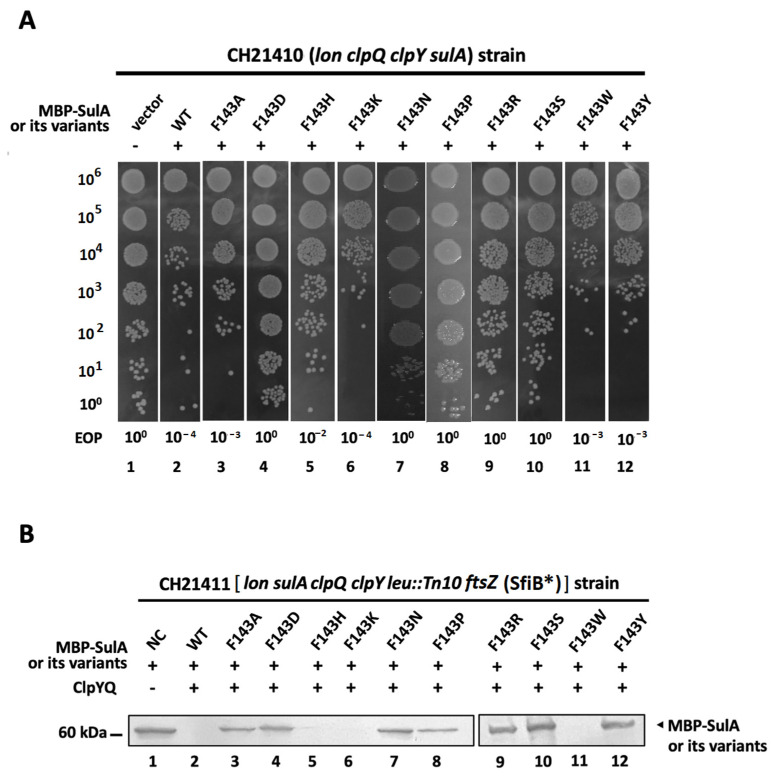
Growth of CH21410 strain with MBP-SulA or its variants and substrate degradation by ClpYQ protease. (**A**) After induction by incubating with 1 mM IPTG for 2 h, bacterial cells expressing MBP-SulA or its variants were diluted in series and spotted on LB media for overnight growth. The EOP value was also determined by using the calculation methods as described in Figure 1C. (**B**) In vivo degradation of MBP-SulA or its variants by ClpYQ protease. CH21411 cells carrying pBAD33-*clpQ^+^* and pBAD24-*clpY^+^* with various pTH18kr-*mbp-sulA^+^* and its derivatives were grown in LB antibiotic with 0.5% L-arabinose to log phase and 1 mM IPTG was added for induction of MBP-SulA or its variants. *ftsZ*(SfiB*) indicates that the genetic disruption is induced by point mutations rather than a complete gene knockout. After 2 h of induction, bacterial cells (2 mL) were collected through centrifugation and cell pellets were re-suspended in 2X-sample buffer and subjected to SDS-PAGE. Equal amounts of each sample protein normalized to OD_600_ were loaded and Western blot analyses were used to detect MBP-SulA or its variants by monoclonal antibody MBP (NEB). An enhanced chemiluminescence (ECL) system from Pierce was used to develop the blots.

To determine the effects of substituting an aromatic or a cation amino acid into the F143 position of SulA on ClpYQ protease degradation, pTH18kr-*mbp-sulA^+^* and its derivative mutation plasmids were separately transformed into the strain CH21410 (*lon*, *clpQ*, *clpY*, *sulA*). After IPTG induction of MBP-SulA and its related derivatives, the resulting transformants were all tested in series for their growth activity. As indicated in Figure 3A, MBP-SulA, SulA^F143K^, SulA^F143W^ and SulA^F143Y^ were lethal to cell growth, with a lower EOP. SulA^F143A^ and SulA^F143H^ possessed moderate lethal activity. However, MBP-SulA^F143D^, SulA^F143N^, SulA^F143P^, SulA^F143R^ and SulA^F143S^ did not affect cell growth, with a higher EOP. As results, an aromatic [Trp (W) and Tyr (Y)] or cation [Lys (K)] residue in the 143rd position of SulA is essential for its lethality. Since the region for the interaction with FtsZ was localized at 84~110 residues in SulA [37], the aromatic ring (F, W and Y), imidazole ring (H) or cation (K) residue in the 143rd position of SulA is likely necessary for its intrinsic stability and a π–π or cation–π interaction might be involved between the structural residues necessary for SulA active activity. To reduce the FtsZ interference in bacterial cells for SulA and its related substrate degradation, *ftsZ* mutations were introduced into CH21410 by means of P1 transduction, and the resultant CH21411 [(*lon*, *clpQ*, *clpY*, *sulA*, *ftsZ*(SfiB*)] strain was created. Then, the above SulA-related plasmids were again separately transformed into the strain CH21411, which carries pBAD33-*clpQ^+^* and pBAD24-*clpY^+^*, and the resulting transformants were tested using in vivo degradation assays. As shown in Figure 3B, ClpYQ protease efficiently degrades MBP-SulA, SulA^F143H^, SulA^F143K^ and SulA^F143W^ at 41 °C. However, it could not efficiently degrade other mutants, including SulA^F143A^, SulA^F143D^, SulA^F143N^, SulA^F143P^, SulA^F143R^, SulA^F143S^ and SulA^F143Y^ (Figure 3B). These physiological results demonstrate that only those SulA mutants with a ring [His (H) and Trp (W)] as well as a cation residue [Lys (K)] at 143rd position were degraded by the ClpYQ protease as efficiently as the wild-type SulA at the higher temperature.

### 2.4. In Vivo Shorter Half-Life of MBP- SulA, SulA^F143H^, SulA^F143K^ and SulA^F143W^ Targeted by the Chromosomal ClpYQ Protease at the Higher Temperature

**Figure 4:**
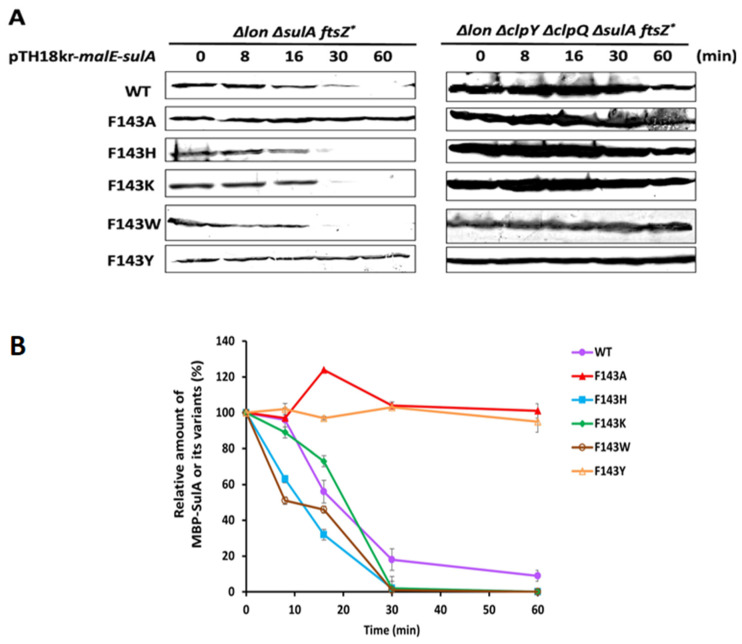
Half-life of MBP-SulA or its variants in the presence or absence of ClpYQ. (**A**) Left panel: Using CH21409 (*lon*, *sulA*, ftsZ*) as the host. Right panel: Using CH21411 (*lon*, *clpY*, *clpQ*, *sulA*, ftsZ*) as the host. Both bacterial strains carrying pTH18kr-*mbp-sulA^+^* or its derivatives were grown at 41 °C to log phase, and spectinomycin (150 μg/mL) was added to the growth media to inhibit synthesis of new proteins. Afterward, MBP-SulA and its variants were quantified and measured at different time intervals. Western blotting with anti-MBP monoclonal antibody was used to detect residual amounts of MBP-SulA and its derivatives at each time point. (**B**) Results from the left panel in (**A**), quantified using Image J, are displayed numerically using the means and standard deviations (error bars) from three independent analyses.

To measure the half-life of wild-type SulA and its related derivatives in bacterial cells, pTH18kr-*mbp^+^-sulA^+^*, *sulA^F143A^*, *sulA^F143H^*, *sulA^F143K^*, *sulA^F143W^* and *sulA^F143Y^* were transformed in series into both CH21409 [(*lon*, *sulA*, *ftsZ*(SfiB*)] and CH21411 [(*lon*, *clpQ*, *clpY*, *sulA*, *ftsZ*(SfiB*)] strains. Since the strain CH21409 [(*lon*, *sulA*, *ftsZ*(SfiB*)] retains an intact *clpQ^+^Y^+^* operon, which is under a heat shock induction, this in vivo degradation assay was thereafter executed at 41 °C. In addition, the residuals of MBP-SulA and its derivative mutants were detected using Western blot assays and their relative levels were determined by using the Image J analyses. As shown, MBP-SulA (18 min), -SulA^F143H^ (12 min) and -SulA^F143W^ (15 min) displayed a shorter half-life (<20 min) and -SulA^F143K^ retained a half-life of approximately 22 min (Figure 4A, the left panel and Figure 4B), indicating that the chromosomally induced ClpYQ protease was capable of degrading the above SulA mutants and the wild-type SulA. Coincidently, they all carry a side-chain ring structure or a cation side group at the 143rd residue. However, both MBP-SulA^F143A^ and -SulA^F143Y^ revealed a longer half-life with extensive stability. These results indicate that SulA mutants, with the side chain of a methyl group or an aromatic ring with a hydroxyl group at the 143rd position, was hardly degraded by ClpYQ (Figure 4A, left panel, and Figure 4B). In addition, to demonstrate that the chromosomal ClpYQ protease is indeed responsible for the above proteolysis of SulA and its mutants, an isogenic strain CH21411, which lacks ClpYQ protease, was subsequently used as a host for measuring the stability of the above SulA and its mutant proteins. As shown, these proteins all physiologically retained prolonged stability (Figure 4A, right panel), indicating that ClpYQ is indeed responsible for the aforementioned intracellular degradations.

### 2.5. In Vitro Pull-Down Analyses between ClpY with MBP-SulA and Its Related Derivatives

**Figure 5:**
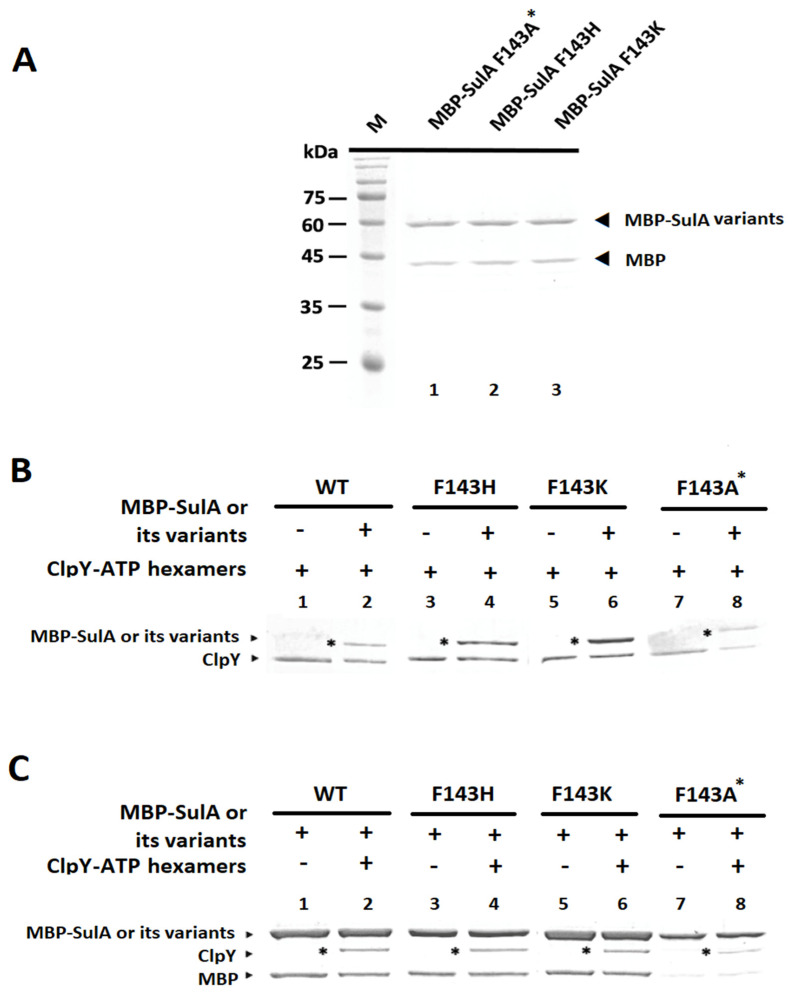
Protein purification and pull-down analyses between ClpY and SulA, as well as its variants. The active MBP-SulA or its variants were purified by amylose resin, which was used to bind maltose-binding protein (MBP) [16,17]. (**A**) Purified proteins of MBP-SulA variants (1 μM) on SDS-polyacrylamide gel. M represents the marker. Lane 1: MBP-SulA^F143A*^ (also with a G110D residue); Lane 2: MBP-SulA^F143H^; Lane 3: MBP-SulA^F143K^. (**B**,**C**) In vitro pull-down assays. Both cobalt and amylose resins were used, respectively, in the pull-down assays. His-ClpY (6-oligomer; 1.3 µM) and MBP-SulA (7.8 µM) were mixed in the pull-down HEPES buffer and then incubated at 41 °C for 3 h in the presence of 5 mM ATP. After incubation, mixtures were separately supplemented with 20 µL of cobalt resin (Takara) and amylose resin (NEB) and rocked at 4 °C for at least 3 h. The resins were then washed 10 times with HEPES buffer (pH 8) to elute off non-binding proteins. Proteins bound to cobalt resin (**B**) or amylose resin (**C**) were separately eluted by 20 µL of 2X SDS-PAGE sampling buffer, subjected to SDS-PAGE, and stained with Coomassie blue R-250.

To analyze binding affinity between ClpY and SulA or its derivatives in the presence of ATP, in vitro pull-down assays were performed after the association of SulA/its derivatives with ClpY_(6x)_-ATP complexes. To achieve this, various MBP-SulA^F143H^, -SulA^F143K^, and -SulA^F143A*^ mutant proteins were separately purified as shown in SDS-PAGE in Figure 5A. Thereafter, using cobalt resin, which conjugates with ClpY, MBP-SulA and its derivatives were individually pulled down in reaction mixtures. Notably, in Figure 5B, ClpY could bind to MBP-SulA, MBP-SulA^F143K^ and MBP-SulA^F143H^ with higher affinity but did not bind well with MBP-SulA^F143A*^. Equally, using amylose resin, which conjugates with MBP-SulA and its related derivatives, ClpY was also pulled down in each sample. Again, in Figure 5C, MBP-SulA, MBP-SulA^F143H^, and MBP-SulA^F143K^ all exhibited higher binding affinity towards ClpY. However, MBP-SulA^F143A*^ exhibited a lower binding activity. These results support the hypothesis that the 143rd residue in SulA would have an effect on the binding affinity towards the hexameric-ClpY.

### 2.6. In Vitro Degradation of MBP-SulA, MBP-SulA^F143H^ and MBP-SulA^F143K^ by ClpYQ, ClpY^Y91F^Q and ClpY^Y91H^Q

**Figure 6:**
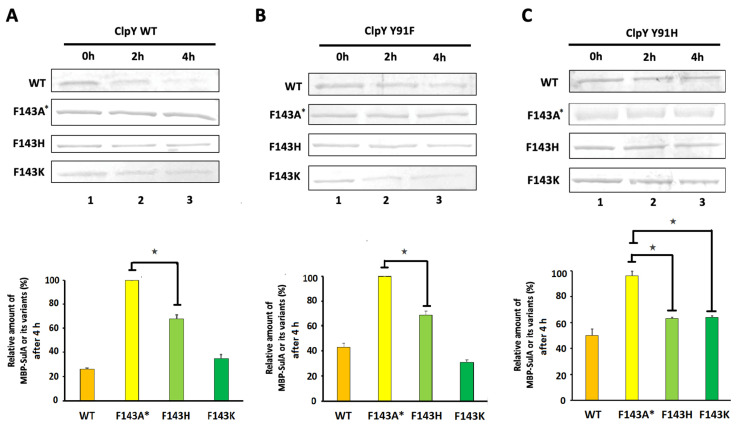
In vitro degradation of MBP-SulA and its variants by ClpYQ, ClpY^Y91F^Q or ClpY^Y91H^Q. MBP-SulA and its variants (1µM) in the presence of ATP (5 mM) and ClpQ (8 µM) were incubated with (**A**) ClpY, (**B**) ClpY^Y91F^ or (**C**) ClpY^Y91H^ in 4µM at 41 °C for 0 h, 2 h and 4 h. In each column, the top panels show the remaining amounts of MBP-SulA and its variants, which were degraded by ClpYQ, ClpY^Y91F^Q or ClpY^Y91H^Q at different time intervals. After 4 h, the relative amounts of residual MBP-SulA and its derivatives were plotted in the bottom chart panels. All results were analyzed in triplicate using Image J. The ★ asterisks indicate *p* < 0.05. Two-way Student’s *t* test for single-pair comparison was used for statistical analysis.

For additional affirmation of our findings, ClpY, ClpY^Y91F^ and ClpY^Y91H^ were in vitro tested individually for their capability to degrade MBP-SulA and its related mutant proteins in the presence of ClpQ and ATP at 41 °C. Notably, wild-type MBP-SulA, MBP-SulA^F143A*^, MBP-SulA^F143H^ and MBP-SulA^F143K^ were each used discretely as a substrate in the degradation assays. Therefore, the residuals of these substrates degraded by ClpYQ or its related derivatives were individually detected from each reaction mixtures by SDS-PAGE at different time points (0, 2 and 4 h). Again, by using Image J analysis, the degradation ratio of MBP-SulA and its related mutants within 4 h were determined. As results, both ClpYQ and ClpY^Y91F^Q proteases could efficiently degrade MBP-SulA and MBP-SulA^F143K^, but moderately degraded MBP-SulA^F143H^ (Figure 6). However, both proteases were not capable of degrading MBP-SulA^F143A*^. Lastly, the ClpY^Y91H^Q protease moderately degraded MBP-SulA, MBP-SulA^F143H^ and MBP-SulA^F143K^, but could not efficiently degrade MBP-SulA^F143A*^. Therefore, these results support the hypothesis that the ring structures of Tyr (Y), Phe (F) and His (H) at the ClpY-91st site are primarily for the functional interaction with the ring structure of Phe (F) and His (H) or the cation structure of Lys (K) in SulA-143rd site of SulA to attain effective substrate degradation by ClpYQ and its derivative proteases.

## 3. Discussion

In our recent studies, we have identified that ClpYQ proteases degrade SulA substrates via recognition of the SulA C-terminal degron site, GFIMRP^147th^ residues [38]. Our data also indicated that the Phe^143rd^ residue of SulA is important for its own native protein-folding. Accordingly, the highly conserved GYVG^93^ sequences constitute the central pore of the hexameric ClpY ATPase [15,16,17]. Specifically, the Tyr^91st^ residue of ClpY plays a vital role in the unfolding/translocation of the substrate proteins for terminal degradation by ClpQ [15,16,17]. Here, we explore the interactive functional roles of the Phe^143rd^ residue of SulA and the Tyr^91st^ residue of ClpY in natural substrate degradation. Since these two important amino acid residues have aromatic rings, we hypothesized that a π–π or an additionally amenable cation–π transient interaction possibly occurs between these two residues and is likely necessary for the recognition and discrimination of substrates by ClpYQ degrader in the processive degradation.

To rigorously explore the unknown mechanisms for SulA degradation by ClpYQ protease, we constructed various point mutations in the ClpY-Tyr^91st^ residue and the SulA-Phe^143rd^ residue. We first conducted a series of physiological tests for the bacteria to demonstrate that the bacterial cells carrying ClpQ with either ClpY, ClpY^Y91F^, ClpY^Y91W^ or ClpY^Y91H^ have lower β-galactosidase activity for *cpsB::lacZ* expression and can survive MMS-induced SulA lethality. These results indicate that the aforementioned ClpY mutants degrade RcsA and SulA proteins. Then, in both in vivo and in vitro degradation assays, ClpY^Y91F^ and ClpY^Y91H^, much like wild-type ClpY, were able to degrade MBP-SulA in the presence of ClpQ. Additionally, ClpY^Y91W^ can minorly degrade MBP-SulA in vitro at a higher temperature. However, from our results, ClpY mutants carrying Y91S, Y91K, and Y91R residues were all defective in their degradation of SulA protein. Similarly, earlier studies demonstrated that ClpY mutants carrying Y91I, Y91C, Y91A and Y91S residues were too defective for SulA degradation [15]. Notably, the F (Phe), W (Trp) and H (His) are aromatic or imidazole amino acids with a ring structure. Therefore, the 91st site of ClpY with a ring structure is important for SulA degradation. Meanwhile, ClpYQ efficiently degrades MBP-SulA, MBP-SulA^F143H^, MBP-SulA^F143W^ and MBP-SulA^F143K^. In addition, all of them possessed the shorter half-life in the intracellular degradation assays. Again, in addition to the ring structure at the 143rd residue adaptable for the substrate degradation, K (Lys) residue that has a cation in the side group can also be apt for the similar degradation. Moreover, in the presence of ClpQ and ATP, both ClpY and ClpY^Y91F^ efficiently degrade MBP-SulA, MBP-SulA^F143H^ and MBP-SulA^F143K^ in vitro. Additionally, under similar conditions, ClpY^Y91H^ also moderately degrades the above substrates. Thus, we conclude that the Phe^143rd^ residue of SulA is functionally involved in a transient π–π interaction with the Tyr^91st^ residue of ClpY in the degradation process. Moreover, an alternative transient cation–π interaction can occur between SulA-143rd and ClpY-91st residues for the substrate processive degradation.

Next, from our in vitro pull-down analyses, ClpY associated well with MBP-SulA, MBP-SulA^F143H^ and MBP-SulA^F143K^, but not with SulA^F143A*^. These results indicate that ClpY was less interactive with SulA^F143A*^ and likely also defective on the unfolding/ translocation of SulA^F143A*^ during degradation. Moreover, after normalizing purified protein levels and quantifying ClpY and MBP-SulA amounts via Image J analyses, we determined the molar ratio for the association between ClpY and MBP-SulA and its derivatives to be 6:1 or 6:2. These results suggest that the ClpY hexamer binds to one or two MBP-SulA molecules and/or its derivatives and also support the hypothesis that ClpYQ degrades its target substrate by means of the translocation of an unfolded polypeptide through the pore site of ClpY-hexamer [18]. In addition, there are two configurations for Y91 residue in the pore site; one is the closed-down state with a functional gripping of the substrate for delivery into the inner region towards the catalytic site, and the other is the opened-up state for slipping in the delivered unfolded substrate for the release [18]. However, all of these studies utilized computational techniques to extrapolate the findings, which still need to be validated through experimental data. Through our studies, we were able to experimentally identify that an initial transient π–π interaction or cation–π interaction is essential for the functional recognition and degradation of a natural substrate by an ATP-dependent protease.

Notably, there are three π–π interaction geometries in aromatic amino-acids: T-shaped, parallel-displaced, and co-facial parallel stacked (sandwich) [42]. In proteins, the former two are the most stable, with nearly iso-energetic symmetry. The last one is the least favorable, with benzene dimers [42]. Both T-shaped and parallel-displaced geometries are electrostatically attractive, yet the direct stacking of aromatic rings is electrostatically repulsive [42]. For our studies, we propose that all three geometries can be adopted for SulA degradation by ClpYQ protease: the 91st-Tyr (Y) of the ClpY hexamer conjugates with SulA at its 143rd-Phe (F) through transient π–π attractive activity, to unfold/translocate SulA into the inner region for further degradation by ClpQ. In the subsequent process, a transient π–π repulsive interaction likely occurs between these ClpY and SulA to release the unfolded protein for the processive degradation. Similarly, a cation–π interaction could be attractive or repulsive between amino acids [53]. Yet, its T-shaped geometry has an interaction stronger than the parallel stacking geometry. Our data indicated that these different geometries could also be applied in SulA^F143K^ degradation by ClpYQ and its derivative degraders. Conversely, the mutants SulA^F143R^ and SulA^F143Y^ may adopt the parallel stacked geometry when their 143rd residue interacts with ClpY-Tyr^91st^ residue, thereby hindering the initial unfolding/translocation action and subsequent degradation process.

Moreover, the cation–π interactions of neutral histidine (His) are attractive, and the cation–π interactions of protonated histidine (His^+^) are repulsive [54]. These propositions also further support that a transient cation–π attractive interaction first occurs between SulA^F143K^ and ClpY^Y91H^ hexamers for the closed-down state, and after translocating substrates into the inner region near the ClpQ catalytic site, a cation–π repulsive interaction for the opened-up state is adopted for the substrate release in the degradation process. We assume that cation–π interactions with a histidine can be reversibly switched between attractive and repulsive interaction under different pH condition, as the histidine becomes protonated and unprotonated. Perceptibly, cation–π interactions affect the two protonation types of histidine. Since the pKa value of histidine changes according to its ambient environment, the amino acid can act as a proton donor or acceptor, in addition to being neutral. Our degradation buffer has a pH value of 7.4, which indicates that the exposed 91st-histidine in the ClpY^Y91H^ hexamer carries a neutral charge and favors an attractive interaction with the cation lysine in SulA^F143K^. This pH value (7.4) also reflects the physiological state of intracellular fluids in bacteria, in which the exposed 91st-histidine in ClpY^Y91H^ also retains the neutral state for the attractive interaction. However, after the unfolding/translocation of the substrates through the pore site into the inner spaces near the ClpQ core site, the 91st histidine residue of ClpY^Y91H^ could be surrounded in a localized acidic environment, which promotes a repulsive interactive activity between SulA-Lys^143rd^ and ClpY-His^91st^ residues to release the unfolded substrate for the progressive degradation.

However, notably, ClpQ exhibits higher catalytic activity in an alkaline environment [24], and the substrate-bound ClpQ at the N-terminal Thr-1 active site could allosterically enhance the ATPase activity of ClpY hexamers and their reciprocal binding affinity [22]. Consequently, it cannot be ruled out that ClpQ, with its catalytic Thr-1 site, might subsequently interact with the unfolded polypeptide in the inner core, facilitating the release of denatured substrate from ClpY for the final cleavage.

Although this study is the first report characterizing the specific π–π or cation–π interaction towards the natural substrate degradation by an ATP-dependent protease, we expect that similar evidence for substrate degradation by other proteases could be found in the future to strengthen the mechanistic model. Other arduous experiments are still needed to explore the molecular mechanisms of substrate degradation by ATP-dependent proteases.

## 4. Materials and Methods

### 4.1. Bacterial Strains, Plasmids and Culture Media

The bacterial strains and plasmids primarily used in this study are listed in Table 1. The primers used here are listed in Supplementary Table S1. The *E. coli* strains were grown in Luria–Bertani (LB) media, if necessary, with the appropriate antibiotics, i.e., ampicillin, 100 μg/mL, kanamycin, 30 μg/mL or chloramphenicol, 20 μg/mL. The pMal-c2X-*sulA^+^* plasmid was kindly gifted by Dr. A. Higashitani [55].

### 4.2. Site-Directed Mutagenesis for ClpY and MBP-SulA Derivative Mutants

A two-step PCR method described previously [17,38] was used to construct each of the ClpY-Tyr^91st^ and SulA-Phe^143rd^ point mutation(s). The PCR reactions were separately performed using either pBAD24-*clpY^+^* or pTH18kr-*malE* (*mbp*)-*sulA^+^* as a template. The primers used in this work are listed in the Supplementary Data. A high-fidelity 2X PCR Master Mix (NEB, Ipswich, MA, USA) was utilized in the PCR reactions. The resultant PCR end products contained *EcoR*I-*Hind*III sites (*clpY^+^* derivatives) for cloning into pBAD24 plasmids and *EcoR*I-*BamH*I sites (*sulA^+^* derivatives) for cloning into pTH18kr-*malE* plasmids, respectively. The acquired plasmids with the correct insertions were confirmed by means of DNA sequencing. Subsequently, the certain DNA fragments derived from pBAD24-*clpY^+^* and pTH18kr-*malE*-*sulA*^+^ constructs were individually ligated into pET21a vectors at the *Nde*I-*Hind*III sites for *clpY* derivatives and into pMal-c2X vectors at the *EcoR*I-*BamH*I sites for *sulA* derivatives.

### 4.3. In Vivo Degradation Assays of MBP-SulA and Its Derivative Mutants

The CH21410 (*lon*, *clpQ*, *clpY*, *sulA*) mutant strain carrying pBAD33-*clpQ*^+^ and pTH18kr-*mbp-sulA^+^* was in-series transformed with pBAD24-*clpY^+^* and its derivatives. At 30 °C, the overnight transformed bacterial cultures were inoculated at 1:100 dilution onto LB media with 0.5% L-arabinose and the appropriate antibiotics. When the growth of bacterial cells reached the mid-log phase, 1 mM IPTG was added. After incubation for about 2 h, 2 mL cell cultures of the samples were collected by means of centrifugation after recording OD_600_. The bacterial cell pellets were then dissolved in 2X SDS-PAGE sample buffer and boiled for 10 min. Each sample normalized to OD_600_ was loaded individually on 12.5% SDS-polyacrylamide gel for electrophoresis. After gel running, the proteins were transferred to the PVDF membrane. Subsequently, MBP-SulA and its derivatives on the PVDF membranes were detected by monoclonal anti-MBP (NEB, Ipswich, MA, USA) with enhanced chemiluminescence (ECL) (Pierce, Rockford, IL, USA) in Western blot assays. In addition, ClpY and its derivatives were detected using a multiple ClpY anti-serum and ClpQ was also detected using a multiple ClpQ anti-serum.

The other in vivo degradation assays were performed via the following procedures. The CH21411 [(*lon*, *clpQ*, *clpY*, *sulA*, *ftsZ* (SfiB*)] mutant strain carrying pBAD33-*clpQ^+^* and pBAD24-*clpY^+^* was serially transformed with pTH18kr-*malE*-*sulA^+^* and its derivatives. Then, the overnight-transformed bacterial cultures were inoculated into LB media with 0.5% L-arabinose and appropriate antibiotics. Subsequently, IPTG (at a final concentration of 1mM) was added after the bacterial cells grew to the mid-log phase. In addition, with an incubation of about 2 h, 2 mL cell cultures of the samples were collected and OD_600_ was recorded. The collected bacterial cell pellets were then dissolved in 2X SDS-PAGE sample buffer with boiling and the dissolved proteins were normalized according to OD_600_ before being loaded on a 12.5% SDS-polyacrylamide gel for electrophoresis and later transferred to PVDF membranes. Afterward, MBP-SulA and its derivatives on the PVDF membranes were detected using methods described above.

The third set of in vivo degradation experiments were performed using the following methods. The CH21409 [(*lon*, *sulA*, *ftsZ* (SfiB*)] and CH21411 [(*lon*, *clpQ*, *clpY*, *sulA*, *ftsZ* (SfiB*)] mutant strains were both serially transformed with pTH18kr-*malE*-*sulA^+^* and its derivatives. The resultant transformed bacterial cells carrying *sulA^+^* or different *sulA* mutant plasmids were then separately grown on LB with kanamycin antibiotic at 41 °C. After the bacterial cells grew to the mid-log phase, IPTG with a final concentration of 1 mM was added to induce protein synthesis for about 1h. Immediately, spectinomycin, with a final concentration of 150 μg/mL, was added to stop the new synthesis of proteins at time 0 and the bacterial cultures were then collected at specific time intervals alongside the recording of OD_600_ reading values. After centrifugation, the bacterial pellets were then dissolved in 2X SDS-PAGE sampling buffers for Western blot assays. Sample loading was normalized according to OD_600_ and equal amounts of the samples were separated by SDS-PAGE before transferring to PVDF membrane. MBP-SulA and its derivatives on the PVDF membranes were detected using the methods described above. The relative amounts of MBP-SulA and its derivatives in bacteria were analyzed using Image J (version 1.45d) [56].

### 4.4. Protein Expression and Purification

The MBP-SulA, His (6x)-tagged ClpY, His (6x)-tagged ClpQ and their mutant variants were each expressed in *E. coli* BL21 (DE3) and purified as described previously [16,17].

### 4.5. In Vitro Degradation Assays of MBP-SulA and Its Derivatives by ClpY or ClpY Mutants in the Presence of ClpQ with ATP

The ClpQ (8 μM), ClpY or its variants (4 μM), MBP-SulA or its variants (1 μM) and ATP (5 mM) were mixed in the degradation buffer composed of 0.1 M HEPES buffer (pH 7.4), 10 mM MgCl_2_, 1 mM dithiothreitol (DTT) and 1 mM EDTA. The reaction mixtures were incubated at 41 °C and the samples were harvested at various time points for analyses of the remaining MBP-SulA and its derivatives. After electrophoresis, the SDS-polyacrylamide gels were stained with Coomassie blue R-250 (Bio-Rad, Hercules, CA, USA) [16,17]. The relative amounts of MBP-SulA and its derivatives were analyzed via Image J (version 1.45d) [56].

### 4.6. In Vitro Pull-Down Assays of ClpY and MBP-SulA or SulA Variants

Amylose resins and cobalt resins were used in pull-down assays. His-ClpY (6-oligomer; 1.3 µM) and MBP-SulA (7.8 µM) were mixed with the pull-down buffer (50 mM HEPES buffer, pH 7.4, containing 150 mM NaCl, 5% glycerol, 5 mM MgCl_2_ and 0.04% Triton X-100) and then incubated at 41 °C for 3 h in the presence of 5 mM ATP [22]. After incubation, the mixtures were supplemented with 20 µL of amylose resins (NEB, Ipswich, MA, USA) or cobalt resins (Takara, Shiga, Japan) and rocked at 4 °C for at least 3 h. The resins were then washed 10 times with 0.2 mL of 50 mM HEPES buffer (pH 8) with 300 mM NaCl, 5% glycerol, 0.04% Triton X-100 and 5 mM MgCl_2_ to remove non-bound proteins. Proteins bound to amylose resins or cobalt resins were separately eluted with 20 µL of 2X SDS-PAGE sampling buffer, subjected to SDS-PAGE, and stained with Coomassie blue R-250 [16,17].

### 4.7. Statistical Analysis

All data were expressed as the mean ± SD of three independent experiments. Statistical analyses were performed using Student’s *t*-test with a significance level of * *p* < 0.05.

## Figures and Tables

**Table 1:** Strains and the primary plasmids used in this study.

Strains	Description	Source and Reference
*E. coli* strains		
BW25113	*lacI^q^ rrnB3 ΔlacZ4787 hsdR514* Δ*(araBAD)567* Δ*(rhaBAD)568 rph-1*	[47]
JW0941	BW25113 *ΔsulA:Kan*	[48]
JW3902	BW25113 *ΔclpY:Kan*	[48]
SG22623	*lon cpsB-lacZ Δ* *ara mal::lacI^Q^*	[49]
CH21408	*lon cpsB-lacZ Δ* *ara mal::lacI^Q^ sulA*	This study
CH21409	*lon cpsB-lacZ Δ* ara mal::lacI^Q^ sulA leu::Tn10 ftsZ(SfiB^*^)	This study
CH21410	*lon cpsB-lacZ Δ* *ara mal::lacI^Q^ clpQ clpY sulA*	This study
CH21411	lon cpsB-lacZ Δara mal::lacI^Q^ clpQ clpY sulA leu::Tn10 ftsZ(SfiB^*^)	This study
Plasmids		
pBAD24	*ori*(pBR322) *ara*C P_BAD_*amp^r^*	[50]
pBAD33	*ori*(pACYC184) *ara*C P_BAD_*cm^r^*	[50]
pTH18kr	*ori*(pSC101) P_lac_*kan^r^*	[51]
pET21a (+)	*ori*(pBR322) T7 promoter *amp^r^*	Novagen
pMAL-c2X	*ori*(pBR322) P_tac_*amp^r^*	NEB
pKD4	Ap Km, *kan* cassette template	[47]
pKD46	*exo*, *bet*, *gam* P_araB_ repA101^ts^ oriR101 *amp^r^*	[47]
pCP20	*FLP^+^* λI1857^+^ λ*P_R_* Rep^ts^ *amp^r^ cm^r^*	[52]

## Data Availability

The data that support the findings of this study are available in the methods and Supplementary Materials of this article.

## Supplementary Materials

The supporting information can be downloaded at: https://www.mdpi.com/article/10.3390/ijms242417353/s1.
