# Supplementary material for: A Transient π–π or Cation–π Interaction between Degron and Degrader Dual Residues: A Key Step for the Substrate Recognition and Discrimination in the Processive Degradation of SulA by ClpYQ (HslUV) Protease in Escherichia coli
Source: Int J Mol Sci. 2023 Dec 11;24(24):17353. doi: 10.3390/ijms242417353 (PMC10743992; doi:10.3390/ijms242417353)
Supplement: Supplementary file 1 [file ijms-24-17353-s001.zip › ijms-2743593-supplementary.pdf]

Supplementary Materials

A Transient π–π or Cation–π Interaction between Degron and Degrader Dual Residues: A Key Step for the Substrate Recognition and Discrimination in the Processive Degradation of SulA by ClpYQ (HslUV) Protease in *Escherichia coli*

Chu-Hsuan Lin [1,†], Chih-Hsuan Tsai [2,†], Chun-Chi Chou [1] and Whei-Fen Wu [1,*]

1 Department of Agricultural Chemistry, College of Bio-Resource and Agriculture, National Taiwan University, Taipei 10617, Taiwan
2 Department of Microbiology and Immunology, College of Medicine, National Cheng Kung University, Tainan 701401, Taiwan
* Correspondence: hfenwu@gmail.com; Tel.: +886-2-3366-4818
† These authors contributed equally to this work.

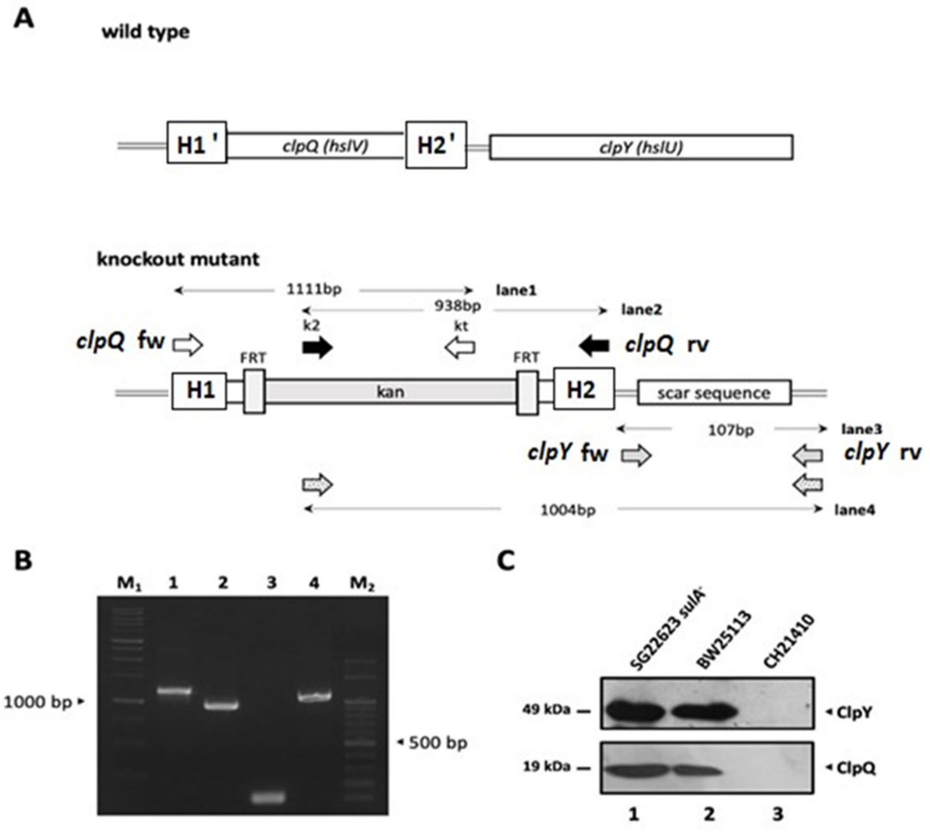

Figure S1. The construction of a new *E. coli* mutant with a deletion of *clpQ*+*Y*+. A JW3902 (*clpY*) mutant strain derived from BW25113, with only the scar sequence inserted in the mutated *clpY* locus from *E. coli* Keio-collection strains [1], was used as the host. The one-step mutagenesis method from Barry Wanner [2] was then adopted to construct a *clpQ* deletion in addition to the *clpY* deletion. (**A**) Illustrations of the whole *clpQ*+*Y*+ genes in the wild-type strain BW25113 and a knockout mutant with a kanamycin-resistant antibiotic marker inserted into the *clpQ* gene in JW3902. As noted, H1 and H2 denote the two designed primers for an insertion of a *kan*^{R} cassette in *clpQ* locus, which is also flanked by FLP recognition target sites. H1' and H2' denote the sequences homologous to the upstream and downstream adjacent site (5'→3' region) to *clpQ*, respectively. Arrow bars represented all the primers used in the PCR reactions to confirm the knock-out mutation in the chromosomal *clpQY* operon. (**B**) PCR products of the above-mentioned primers that were separated by agarose gel (1%) electrophoresis. Afterward, the chromosomal *Kan*^{R} cassette in bacteria was removed by the plasmids

pCP20, which could express the specific recombinase to act at the FLP sites. pCP20 itself was latter removed from bacteria while growing at 37ºC. (C) The Western blotting analyses for the *clpQY* knock-out mutant CH21410. The BW25113 and SG22623 *sulA* strains were used as the controls. The multi-serum antibodies of ClpQ and ClpY were used for detecting the *clpQY* deletions in bacteria.

Table S1. Primers used in this study.

Primers [a]	DNA Sequence (5'→3')	Note
clpQ mutant Fw (H1)	TTTGTACTCTGTATTCGTAACCAAGGGGTCAGCTCGTGACAACTATAGTA*TGTGTAGGCTGGAGCTGCTT*	b
clpQ mutant Rv (H2)	*TTACGCTTTGTAGCTT*AATTCTTCGATGGTGTGGAAATGGTTGGTATAGAT*CATATGAATATCCTCCTTAG*	b
k2	CGGTGCCCTGAATGAACTGC	c
kt	CGGCCACAGTCGATGAATCC	c
clpQ fw	CGTAACCAAGGGGTCAGCTCGTGACAACTATAGTA	c
clpQ rv	TTACGCTTTGTAGCTTAATTCTTCGATGGTGTGG	c
clpY fw	ATGATTCCGGGGATCCGTC	c
clpY rw	CGCGATTATAGGATAAAACGGC	c
EcoRI sulA Fw	CCGGAATTCATGTACACTTCAGGCTATGCACATC	d
sulA F143H Rv	CGCATAAT**ATG**CCCCATAGCGTTACCTTCATTTGCCGC	e
sulA F143K Rv	CGCATAAT**TTT**CCCCATAGCGTTACCTTCATTTGCCGC	e
sulA F143P Rv	CGCATAAT**AGG**CCCCATAGCGTTACCTTCATTTGCCGC	e
sulA F143R Rv	CGCATAAT**GCG**CCCCATAGCGTTACCTTCATTTGCCGC	e
sulA F143W Rv	CGCATAAT**CCA**CCCCATAGCGTTACCTTCATTTGCCGC	e
sulA BamHI Rv	CGCGGATCCTTAATGATACAAATTAGAGTGAATTTTTAG	d
sulA F143H Fw	CTATGGGG**CAT**ATTATGCGTCCGGTAAGCGCATCCT	e
sulA F143K Fw	CTATGGGG**AAA**ATTATGCGTCCGGTAAGCGCATCCT	e
sulA F143P Fw	CTATGGGG**CCT**ATTATGCGTCCGGTAAGCGCATCCT	e
sulA F143R Fw	CTATGGGG**CGC**ATTATGCGTCCGGTAAGCGCATCCT	e
sulA F143W Fw	CTATGGGG**TGG**ATTATGCGTCCGGTAAGCGCATCCT	e
NdeI-His-*clpY* Fw	GGAATTCCATATGCACCACCACCACCACCACTCTGAAATGACCCCACGC	d
EcoRI- clpY Fw	CCGGAATTCATGTCTGAAATGACCCCACGC	d
clpY Y91F Rv	TTACCGAC**AAA**GCCCACTTCGGTGAATTTGGTCGCTTC	e
clpY Y91H Rv	TTACCGAC**GTG**GCCCACTTCGGTGAATTTGGTCGCTTC	e
clpY Y91K Rv	TTACCGAC**TTT**GCCCACTTCGGTGAATTTGGTCGCTTC	e
clpY Y91R Rv	TTACCGAC**GCG**GCCCACTTCGGTGAATTTGGTCGCTTC	e
clpY Y91S Rv	TTACCGAC**GGA**GCCCACTTCGGTGAATTTGGTCGCTTC	e
clpY Y91W Rv	TTACCGAC**CCA**GCCCACTTCGGTGAATTTGGTCGCTTC	e
clpY HindIII Rv	CCCAAGCTTTTATAGGATAAAACGGCTCAGA	d
clpY Y91F Fw	AAGTGGGC**TTT**GTCGGTAAGGAAGTGGATTCTATTA	e
clpY Y91H Fw	AAGTGGGC**CAC**GTCGGTAAGGAAGTGGATTCTATTA	e
clpY Y91K Fw	AAGTGGGC**AAA**GTCGGTAAGGAAGTGGATTCTATTA	e
clpY Y91R Fw	AAGTGGGC**CGC**GTCGGTAAGGAAGTGGATTCTATTA	e
clpY Y91S Fw	AAGTGGGC**TCC**GTCGGTAAGGAAGTGGATTCTATTA	e
clpY Y91W Fw	AAGTGGGC**TGG**GTCGGTAAGGAAGTGGATTCTATTA	e

[a] Fw represents the forward primer and Rv represents the reverse primer. [b] The italic characteristics indicate the nucleotide sequences in homology with those adjacent to the target *kanamycin* cassette at either side (5'→3' region) in pKD4 plasmids. [c] The primers used in PCR reactions for the DNA fragments correctly identified in the *clpYQ* knock-out operon mutant. [d] The underlines indicate the restriction enzyme cutting sites. [e] The bold characteristics indicate the mutated nucleotides.

References

1. Baba, T.; Ara, T.; Hasegawa, M.; Takai, Y.; Okumura, Y.; Baba, M.; Datsenko, K.A.; Tomita, M.; Wanner, B.L.; Mori, H. Construction of *Escherichia coli* K-12 in-frame, single-gene knockout mutants: The Keio collection. *Mol. Syst. Biol.***2006**, *2*, 2006.0008.
2. Datsenko, K.A.; Wanner, B.L. One-step inactivation of chromosomal genes in *Escherichia coli* K-12 using PCR products. *Proc. Natl. Acad. Sci. USA* **2000**, *97*, 6640–6645.
